# Congenital Portosystemic Shunts in Dogs and Cats: Treatment, Complications and Prognosis

**DOI:** 10.3390/vetsci10050346

**Published:** 2023-05-12

**Authors:** Alexandros O. Konstantinidis, Katerina K. Adamama-Moraitou, Michail N. Patsikas, Lysimachos G. Papazoglou

**Affiliations:** 1 Companion Animal Clinic (Medicine Unit), School of Veterinary Medicine, Faculty of Health Sciences, Aristotle University of Thessaloniki, 54627 Thessaloniki, Greece; kadamama@vet.auth.gr; 2 Laboratory of Diagnostic Imaging, School of Veterinary Medicine, Faculty of Health Sciences, Aristotle University of Thessaloniki, 54627 Thessaloniki, Greece; patsikm@vet.auth.gr; 3 Companion Animal Clinic (Surgery and Obstetrics Unit), School of Veterinary Medicine, Faculty of Health Sciences, Aristotle University of Thessaloniki, 54627 Thessaloniki, Greece; makdvm@vet.auth.gr

**Keywords:** attenuation, canine, complications, feline, portosystemic shunt, prognosis, treatment

## Abstract

**Simple Summary:**

Congenital portosystemic shunts (CPSS) are anomalous vessels connecting the portal vein, or its tributaries, with the systemic circulation. CPSS permit venous blood, draining from the spleen, pancreas, and major areas of the gastrointestinal tract, to bypass the liver and directly enter the systemic circulation. They are either extrahepatic (ECPSS) or intrahepatic (ICPSS), single or multiple. ECPSS are most common within small breed dogs such as Maltese, Yorkshire terriers, and Poodles, while ICPSS are most common within large breed dogs such as Irish wolfhounds, and Labrador retrievers. However, they are rare in cats. Clinical signs of CPSS are non-specific and may wax and wane, while laboratory findings can raise the clinical suspicion for CPSS, but they are also not specific. Definitive diagnosis will be established by evaluation of liver function tests and diagnostic imaging. Attenuation of the CPSS is the treatment of choice and may be performed by open surgical intervention using ameroid ring constrictors, thin film banding, and partial or complete suture ligation or by percutaneous transvenous coil embolization. Medical management of dogs and cats with CPSS is indicated pre-surgically when stabilization is required, or when surgery is not possible. Medical treatment strategies include administration of non-absorbable disaccharides (i.e., lactulose), antibiotics, and dietary changes. After CPSS attenuation, short- and long-term post-surgical complications may be seen, such as post-operative seizures and recurrence of clinical signs, respectively. Prognosis after surgical attenuation of CPSS is generally favorable for dogs and fair for cats.

**Abstract:**

Congenital portosystemic shunts (CPSS) are a common vascular anomaly of the liver in dogs and cats. Clinical signs of CPSS are non-specific and may wax and wane, while laboratory findings can raise the clinical suspicion for CPSS, but they are also not specific. Definitive diagnosis will be established by evaluation of liver function tests and diagnostic imaging. The aim of this article is to review the management, both medical and surgical, complications, and prognosis of CPSS in dogs and cats. Attenuation of the CPSS is the treatment of choice and may be performed by open surgical intervention using ameroid ring constrictors, thin film banding, and partial or complete suture ligation or by percutaneous transvenous coil embolization. There is no strong evidence to recommend one surgical technique over another. Medical treatment strategies include administration of non-absorbable disaccharides (i.e., lactulose), antibiotics, and dietary changes, and are indicated for pre-surgical stabilization or when surgical intervention is not feasible. After CPSS attenuation, short- and long-term post-surgical complications may be seen, such as post-operative seizures and recurrence of clinical signs, respectively. Prognosis after surgical attenuation of CPSS is generally favorable for dogs and fair for cats.

## 1. Introduction

Congenital portosystemic shunts (CPSS) are abnormal vascular communications between the portal and the systemic circulation, bypassing the hepatic sinusoids and parenchyma [1,2]. CPSS can be either extrahepatic (ECPSS) or intrahepatic (ICPSS). ECPSS are most common within small breed dogs such as Maltese, Yorkshire terriers, and Poodles, while ICPSS are most common within large breed dogs such as Irish wolfhounds, and Labrador retrievers [3,4,5,6,7,8]. While these abnormalities are common in dogs, they are rare in cats [9,10]. Neurological abnormalities due to hepatic encephalopathy (HE) are usually the most common clinical signs at presentation, although gastrointestinal and urinary tract signs are also frequently reported [3,11]. Surgical attenuation of CPSS is the recommended treatment for most cases in order to restore normal portal blood flow and resolve clinical signs [2,9,12,13,14]. A variety of surgical techniques for attenuation of CPSS, acute or gradual, have been proposed including suture ligation, ameroid ring constrictor (AC), thin film banding (TFB), hydraulic occluder (HO), and self-retaining polyacrylic acid-silicone device placement and intravascular techniques [percutaneous transvenous coil embolization (PTCE)]. Medical management of dogs and cats with CPSS is indicated pre-surgically when stabilization is required, or when surgery is not possible. The long-term survival of dogs with CPSS treated surgically is greater than those managed medically.

The purpose of this article is to review the treatment of CPSS, medical and surgical, their complications, as well as the prognosis. Moreover, a comparison among selected surgical techniques will be attempted according to the knowledge gathered from the literature and authors’ experience.

## 2. Medical Management of Congenital Portosystemic Shunts

Medical management of dogs and cats with CPSS is required pre-surgically for patient stabilization, post-surgically especially in dogs and cats with insufficient clinical improvement, when surgical correction is not possible due to shunt location or type, or due to owner’s denial for any surgical correction [12,13,15]. Medical therapy aims to reduce intestinal production and absorption of encephalopathic toxins from the gastrointestinal (GI) tract. However, medical management only controls clinical signs and does not treat underlying pathology. Medical management mainly includes nutritional support/ dietary adjustments and administration of lactulose and antibiotics. A minimum 2-week stabilization period with medical management is recommended before CPSS attenuation [1,16]. Surgical attenuation of the shunt is generally recommended for dogs with CPSS because of improved survival and quality of life compared to dogs treated medically [12,17].

Nutritional support of dogs and cats with CPSS is very important [1,18]. These animals should be fed a complete and balanced, highly palatable, and highly digestible diet that contains the appropriate type and quantity of proteins and is supplemented with all the essential vitamins and minerals. Dogs and cats with CPSS without clinical signs of HE should not undergo severe protein restriction (especially those with poor body condition), as it can lead to increased muscle catabolism promoting further hyperammonemia [19,20]. On the contrary, they should be fed as much protein as they will tolerate without becoming clinically encephalopathic [19,21]. The goal is the diet to contain 18–22% proteins for dogs and 30–35% for cats on dry matter basis [1]. Non-meat protein-based diets (e.g., dairy- or vegetable-based protein diet) are often recommended for dogs with HE [19,20]. Commercially prepared prescription diets for liver support are appropriate for protein restriction in patients with HE [18,20,22,23,24]. Renal clinical diets should be avoided, as severe protein restriction is not recommended pre-surgically or for long-term management for all patients [24]. However, in cases with severe HE, dietary protein restriction is required, and therapeutic renal diets can be used short term. Small and frequent meals help the patient minimize the clinical signs associated with HE [18]. Monitoring weight, body and muscle condition score, and serum albumin levels is recommended [18,20]. Patients that underwent shunt attenuation should typically slowly return to maintenance diets usually within 2–3 months post-operatively [8,24].

Lactulose is a non-absorbable synthetic disaccharide, administered orally or as an enema, and is the main therapy for decreasing absorption of ammonia and other neurotoxins [1,18,22]. Lactulose is metabolized by colonic bacteria to organic acids (i.e., lactic, acetic, and formic acid), which increase osmotic pressure drawing water into the bowel and acidify colonic contents [25]. Acidification of the colonic contents leads to the conversion of ammonia to non-absorbable ammonium and alters colonic microbiota by inhibiting the growth of ammonia-producing bacteria. [25]. Alteration of intestinal transit time associated with the osmotic diarrhea decreases the available time for ammonia production and absorption [18,26]. The initial dose of lactulose is low and gradually increases until achieving several soft stools per day (Table 1) [18,22]. Lactitol is another non-absorbable synthetic disaccharide in powder form but not available worldwide. Non-absorbable disaccharides should be used with caution. Common side effects in high doses are diarrhea, vomiting, anorexia, increased GI loss of potassium and water, and abdominal cramping [27].

If there is no adequate response to diet modification and lactulose, oral administration of ideally non-absorbable or poorly absorbable antibiotics is required to change the colonic microbiota by decreasing the urease-producing bacteria and subsequently decreasing ammonia production and absorption [28]. Metronidazole, neomycin, and ampicillin have been used in small animals with HE (Table 1) [15,18,22]. Neomycin, although poorly absorbed from the GI tract, is no longer recommended due to its nephrotoxic and ototoxic effects. Metronidazole undergoes extensive hepatic metabolism and the dose must be reduced in dogs and cats with HE due to CPSS (Table 1). Studies regarding the use of metronidazole in treatment of HE are limited in veterinary medicine. In a very recent study, Serrano et al. (2022) compared the effect of diets for liver support, lactulose, and metronidazole in pre-surgical stabilization in dogs with ECPSS [29]. Metronidazole did not have additional benefits, and the combination of liver support diets with lactulose provided appropriate control of clinical signs in the studied population of dogs with ECPSS awaiting surgical attenuation. Finally, rifaximin, a semisynthetic non-absorbable derivative of rifampicin, is effective at treating and preventing HE in humans [30]. Rifaximin has been proved more effective in lowering blood ammonia levels and improving clinical signs associated with HE compared to neomycin [31]. There are no studies currently examining the efficacy of rifaximin in dogs with HE.

Dogs with ICPSS have a predisposition to develop GI ulceration both pre- and post-operatively [5]. When gastroduodenal ulceration or erosion is present or is suspected, administration of gastroprotectants, especially proton pump inhibitors (e.g., omeprazole), is recommended (Table 1). Sucralfate may also be used [1,22].

Dogs and cats with CPSS may present with an acute crisis of HE, demonstrating severe neurological signs such as seizures, lethargy, or coma [3,32,33,34]. Any animal with an exacerbation of clinical signs should be thoroughly investigated for precipitating factors such as dehydration, high-protein meals, gastrointestinal hemorrhage, uremia, constipation, and sepsis [18,22]. Any concurrent drug therapy should be reviewed for the potential to cause dehydration, electrolyte imbalances, or hepatotoxicity. Therapy for acute and severe exacerbation of chronic HE includes administration of lactulose per rectum after a cleansing warm water enema, and antibiotics to decrease urease-producing bacteria (metronidazole, ampicillin, or amoxicillin) (Table 1) [18,22]. Anticonvulsants should be also administered to CPSS patients with seizures due to HE pre- and/or post-operative (POS) seizures. Many clinicians try to control seizures using low dose of midazolam. The use of benzodiazepines, such as diazepam and midazolam, to control seizures due to HE is controversial, and there are no clinical trials that have evaluated the efficacy and side effects of these drugs. In humans, benzodiazepine administration is considered to be a precipitating factor for HE [35]. Administration of levetiracetam, phenobarbital, propofol, and potassium bromide may also be considered.

In humans, few studies have suggested that activation of the gamma-amino-butyric-acid (GABA)/benzodiazepine inhibitory neurotransmitter system contributes to HE pathogenesis [36,37,38]. Flumazenil, an antagonist of the centrally acting gamma-aminobutyric acid (GABA) receptors, exerts its mechanism of action by competitively inhibiting the benzodiazepine site on the GABA receptor [39]. In a metanalysis conducted by Goulenok et al. (2002), it was shown that flumazenil induces clinical and electroencephalographic improvement of HE in patients with liver cirrhosis [40]. However, the evidence was graded to be of low quality. At present, evidence for the beneficial short-term efficacy of flumazenil in treating humans with HE is limited. It probably has a small benefit in acute episodes of encephalopathy, especially in situations of benzodiazepine intoxication/overdose. In dogs, the efficacy of flumazenil was evaluated by Meyer et al. (1998) in a group of dogs with chronic HE due to Eck fistula [41]. These dogs did not respond to flumazenil; this finding suggests that endogenous benzodiazepines are unlikely to play a significant role in the pathogenesis of canine HE.

## 3. Surgical Treatment of Congenital Portosystemic Shunts

Surgical correction of CPSS aims at shunt attenuation and re-establishment of normal blood flow to the hepatic parenchyma. Successful occlusion of the shunt will result in the development of the portal vasculature and increase in the liver volume [42,43,44,45,46]. CPSS can be ligated partially or completely using non-absorbable sutures or gradually attenuated using an AC, TFB, or HO [3,47,48,49,50,51,52]. The majority of dogs and cats with CPPS cannot tolerate acute complete attenuation [53,54,55]. Surgical treatment of ICPSS is much more complex compared to that of ECPSS, due to the location of the shunt within the liver parenchyma [56,57]. Surgical methods for gradual attenuation of the shunt were developed to limit life-threating complications after acute complete attenuation of CPSS. Computed tomography angiography (CTA) is a valuable, safe, fast, and accurate diagnostic imaging method not only for diagnosis of CPSS but also for planning of surgery [58,59]. CTA offers great morphological characterization (origin, insertion, and diameter) of the CPSS. These advantages of CTA offer significantly reduced operative time and should be performed pre-surgically if available. Liver biopsies could be obtained as a basis for future comparison, even if histological changes are not associated with prognosis [60].

### 3.1. Suture Ligation

**Figure 1:**
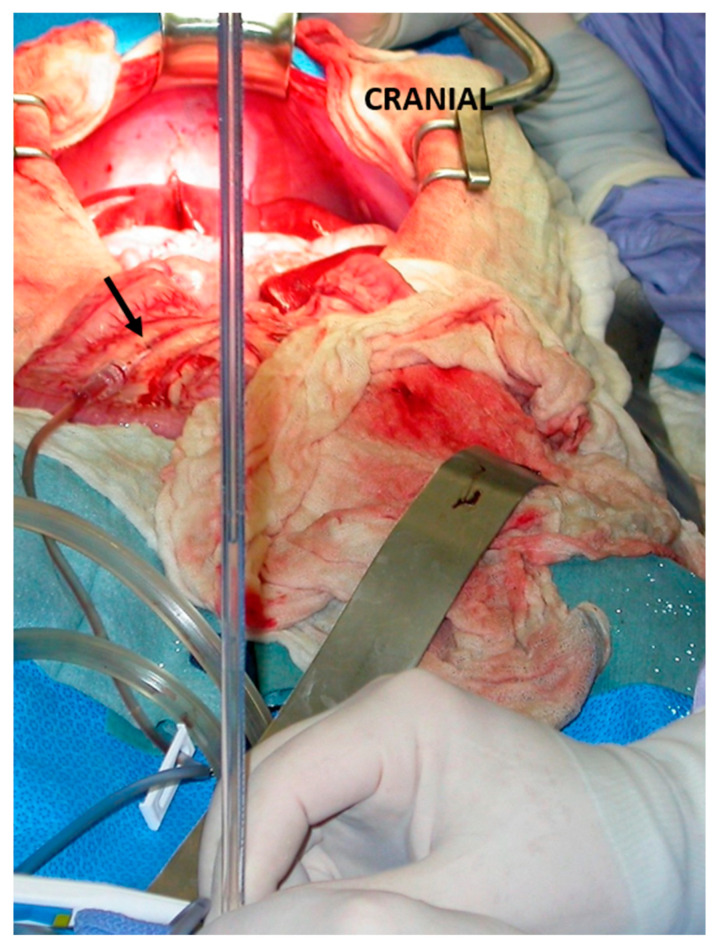
Intraoperative measurement of portal pressure through a mesenteric vein catherization (arrow) during an intrahepatic shunt attenuation in a dog.

Suture ligation is the first method described for CPSS attenuation using silk or polypropylene suture material. Acute complete or partial occlusion of CPSS can be performed; however, the majority of dogs (≈50–80% of dogs with ECPSS and ≈82–85% of dogs with ICPSS) and cats (57–71%) with a CPSS do not tolerate acute complete occlusion due to the insufficient portal vasculature and development of portal hypertension (PH) [42,49,52,53,54,55,57,61,62,63,64,65]. Intraoperatively, the portal pressure is measured to determine if complete ligation is possible, via a jejunal, splenic, or a portal vein catheter prior and after temporary shunt occlusion. Post-ligation portal venous pressure greater than 17 to 24 cm water and an increase in portal pressure greater than 9 to 10 cm water is associated with a negative outcome [4,53,66,67,68] (Figure 1). The CPSS must be ligated to a point of pressure between these values. Central venous pressure should also be monitored during shunt occlusion. A decrease greater than 1 cm water in central venous pressure was associated with post-operative PH [53]. Blood pressures can vary with the depth of anesthesia, hydration status, phase of respiration, degree of splanchnic compliance, and other systemic factors and should also be considered [53,68,69,70,71]. Post-ligation pallor or cyanosis of the intestines, increased intestinal peristalsis, increased mesenteric vascular pulsations and cyanosis, or edema of the pancreas are evidence of PH and can be also used as criteria for the tolerated degree of attenuation [72]. In suture ligation, complete shunt attenuation is desirable as it has been correlated with better outcome [11]. Subsequently, a second surgery for complete ligation for animals undergoing partial ligation of CPSS (ICPSS or ECPSS) is usually required, as complete ligation has been associated with a better long term outcome [17,43,53,54,55,57,65,73]. Staged suture ligation may ensure complete attenuation of the shunt, and reduce the chance of recurring clinical signs; however, acquired portosystemic shunts formation is still possible [43,54,65]. In a recent study including 55 dogs with ICPSS, only 18.2% tolerated complete attenuation [65]. However, the majority of dogs that did not tolerate complete attenuation underwent a second surgery, and complete attenuation in two surgeries was achieved in 27/33 (81.8%) of dogs [65]. In another recent study, complete ligation was possible in 76% of dogs with ECPSS [74]. Interestingly, liver function tests return to normal in a number of dogs undergoing single partial ligation indicating that shunts may continue to narrow after initial attenuation [55,73,75].

### 3.2. Gradual Attenuation

**Figure 2:**
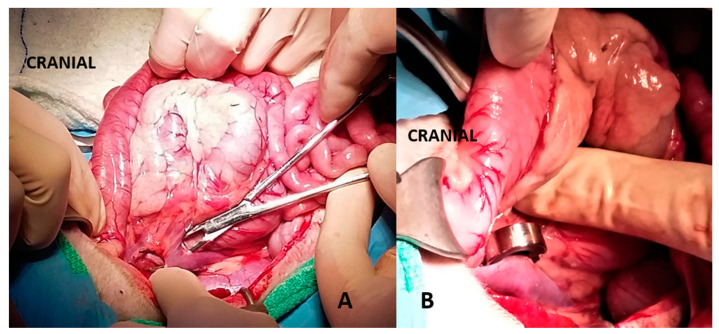
(**A**): A portocaval shunt is evident over a right angle forceps. (**B**): An ameroid constrictor was placed around the shunt for attenuation.

**Figure 3:**
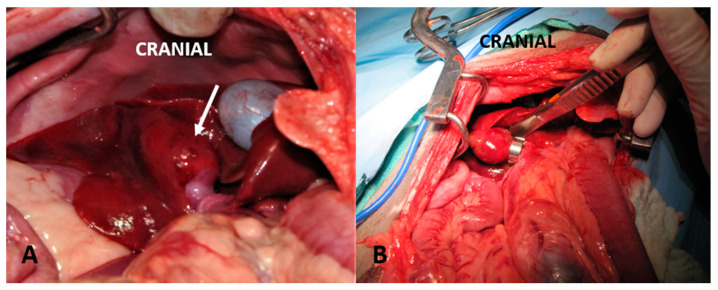
(**A**): Intrahepatic portosystemic shunt of the caudate lobe creating an aneurism within the hepatic paranchyma (arrow). (**B**): Ameroid constrictor placed around the right portal branch that supplies the caudate lobe.

Gradual occlusion of the CPSS allows re-establishment of the hepatic architecture due to the gradually increased vascular supply and at the same time avoiding fatal PH. AC or TFB are almost exclusively used for gradual CPSS occlusion. AC are devices offering gradual shunt occlusion and can be used for both ECPSS and ICPSS. They have an inner ring of compressed casein that is surrounded by a stainless-steel sheath [76]. The casein ring of the AC expands slowly after implantation as it absorbs body fluid, resulting to shunt occlusion due to compression, fibrosis, and thrombosis formation causing gradual occlusion within 2–5 weeks after placement [1,45,50,76] (Figure 2 and Figure 3). Closure is fast the first 3–14 days, after implantation, and declines after the ring internal diameter has been reduced by 32% [47]. Use of AC reduces the risk of PH by allowing the hypoplastic portal vasculature time to adopt to the increased blood flow [76], the overall surgical time, and probably the overall cost compared to suture ligation [53,76,77,78]. Despite the gradual occlusion of the CPSS after AC placement, acquired portosystemic shunts development has been reported in 40% of dogs with ICPSS and in 17% of dogs with ECPSS [3,63,76].

**Figure 4:**
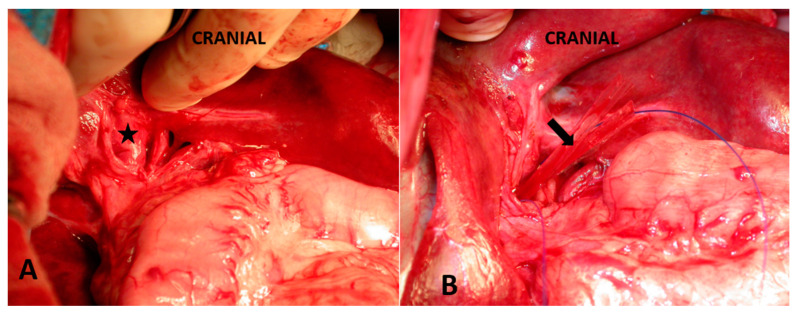
(**A**): A right divisional portosystemic shunt (asterisk) is visualized. (**B**): A thin film (arrow) was placed around the shunt. A polypropylene suture that was placed around the shunt to facilitate easier thin film placement was removed prior to celiotomy closure.

TFB is an alternative to ACs for gradual occlusion of CPSS. Similar to ACs, TFB cause fibrous tissue reaction and gradual shunt occlusion [50] (Figure 4). They are constructed from non-medical general usage cellophane. The film is cut into strips (1–1.2 cm × 10 cm) and gas sterilized [1,79]. The thin film bands are secured around the shunt with vascular clips. Use of TFB for CPSS attenuation was initially suggested by Breznock (1979) but it was firstly used by Harari et al. (1990) [66,80]. They placed successfully 3 mm wide thin films around a portoazygous CPSS occluding the shunting vessel by approximately 50% [66,80]. Since then, TFB has been used for the attenuation of CPSS in dogs and cats [51,81,82,83,84,85]. Initially, attenuation of the shunt to less than 3 mm was performed [51]. However, Frankel et al. (2006) showed that complete occlusion of the shunt was possible without larger than 3 mm attenuation even for dogs with ECPPS [48]. Persistent shunt flow after TFB has been reported in cats and is possibly due to reduced inflammatory response in this species [51,86,87]. However, in a recent retrospective case series, of 34 cats with ECPSS treated by application of TFB, serum bile acid (BA) concentrations normalized in 25 of 28 of the cats post-surgically, and only 1 cat with abnormal BA had a patent shunt at the time of a second exploratory surgery [88]. A variation of TFB, using polyolefin fiber thin film, led to similar to AC long-term outcomes in dogs with ECPSS in a recent large retrospective study [89].

HO is an inflatable silicone and polyester cuff connected by a tube to an access port placed under the skin. The HO is placed around a vessel (as an AC) and is maintained in position with a non-absorbable suture. Post-surgically, HO is inflated using small amounts of sterile saline through the access port. HO allows complete and progressive occlusion of the shunt that can be personalized to each patient needs (clinical signs and serum biochemistry). However, HO usage for shunt occlusion is limited [47]. Ten dogs, with left-, central-, and right- divisional ICPSS, were treated by applying a HO around the portal branch supplying the shunt [47]. A small amount of sterile saline was injected in the port of HO every 2 weeks and shunt closure occurred in 6–8 weeks. In 3/10 dogs, HO was ruptured showing elevated post-prandial serum BA, confirming the impression that HO cannot induce inflammation and closure of the shunt in the long term. In 2/10 dogs, ascites after surgery delayed inflation of the cuff until ascites resolved.

The use of a self-retaining polyacrylic acid-silicone device is another very promising option for ECPSS attenuation [90]. This device closes the shunt via gradual physical occlusion over a 6–8-week period without relying on inflammation, fibrosis, or thrombosis as with attenuation by a AC or TFB. The advantages of this device are the consistency in closure times and the ease of application. In a prospective clinical trial, this device led to complete occlusion by 8 weeks in 4/6 of dogs, with the remaining 2/6 having only mild residual flow [90]. However, this device is not yet commercially available.

### 3.3. Percutaneous Transvenous Coil Embolization

Spreading of interventional radiology in small animal surgery offered PTCE as an alternative technique for the occlusion of ICPSS but also of ECPSS in dogs and cats [33,82,91,92,93,94,95]. PTCE is a minimally invasive, fast, and promising procedure for the occlusion of ICPSS that could reduce PH and splanchnic congestion incidence as well as high mortality rates of open surgical techniques but requires further evaluation. The coils used in PTCE are flexible metallic strips with multiple polyester fiber. These coils are placed, under fluoroscopic guidance, into the vessel lumen. Coils primarily cause reduction in the shunt flow and secondary form a thrombus; occlusion occurs within 1–2 months. While coils are placed in the CPSS, portal pressure is monitored [5]. Additional coils can be added in the future if patient’s clinical signs persist or the patient cannot be weaned from medication [5]. The two most important complications of PTCE are PH due to rapid thrombosis and coil migration. Partington et al. (1993) performed four separate embolization procedures in a dog with ICPSS in order to achieve gradually occlusion of the shunt and reformation of the intrahepatic portal perfusion [91]. Usage of non-fibered coils, maintaining some flow through the shunting vessel, or administration of antithrombotic treatment are alternative choices to prevent fatal PH [91,96]. Coil migration to the heart or lungs is another possible complication of PTCE [91,97,98] due to the high flow rates or shunt large diameter. Stent placement to the entrance of the shunt in the caudal vena cava or the hepatic vein prevents migration of coils due to high flow rates [94,96,97,98]. However, no recommendations of one treatment over another can be made at the moment because of the paucity of evidence of clinical outcomes in dogs with ECPSS or ICPSS [17,65,99,100].

## 4. Complications after Congenital Portosystemic Shunt Attenuation

PH is most commonly seen in dogs undergoing acute suture ligation and less commonly in dogs undergoing gradual shunt occlusion [48,51,53,54,55,57,77,78,101]. Clinical signs of acute severe PH include abdominal pain and distention due to ascites or ileus, hypovolemic shock, vomiting, and diarrhea containing fresh or digested blood due to GI hemorrhage [1,2]. In cases of mild or moderate PH, the only clinical sign is ascites [1,2]. Dogs with acute severe PH should be supported with crystalloids, analgesics, gastrointestinal protectants, and warmth in case of hypothermia as it is a negative prognostic factor post-operatively [4]. In case of severe abdominal distention and dyspnea, spironolactone and/or furosemide are usually administered. Overhydration, external abdominal compression, and large meals should be ideally avoided [102].

Post-operative neurological signs (PONS) are a common complication of the surgical management of CPSS reported in 3.6–12% of dogs in 36.7–60% of cats [5,42,52,72,101,103,104,105,106,107,108]. Signs vary from mild ataxia, depression, and disorientation to generalized seizure activity [63,72,104,106,109,110]. Post-operative seizures (POS) are a frequently fatal complication reported up to 8% of dogs and 23.5% of cats after shunt attenuation in recent studies [52,88,103,105,109,111]. They are most common in small breed dogs with ECPSS but have been reported also after attenuation of ICPSS and occur up to 3 days post-operatively [112]. Sometimes, other neurological symptoms including ataxia, depression, disorientation, vocalization, blindness, and muscle tremor are present before generalized seizure activity occurs [104]. The pathogenesis of POS is unknown but potential etiologies include decrease in systematic concentrations of endogenous benzodiazepines, imbalance in excitatory and inhibitory neurotransmitters, hypoglycemia, HE, hypoxemia, systemic hypertension, electrolyte disturbances, and concurrent brain disease [104,106,112,113,114,115,116], although it has been shown that POS are not associated with hypoglycemia, hyperammonemia, or electrolyte derangement [106,117]. Pre-operative treatment with anticonvulsant drugs has been proposed to decrease the risk of POS. However, POS incidence did not decrease after potassium bromide administration 2 weeks before surgery [3]. Similarly, administration of phenobarbital did not significantly decrease post-operative neurological dysfunction but may have prevented development of generalized motor seizures or status epilepticus [104]. There are conflicting results regarding the pre-operative administration of levetiracetam (20 mg/kg PO q8h for a minimum of 24 h) to reduce the probability of POS [105,111,116]. Benzodiazepines, barbiturates, and propofol (as a bolus or CRI) have been used to control status epilepticus with conflicting results [72,104,106,117,118]. It is always important to rule out hypoglycemia, HE, and electrolyte disorders as seizure causes. POS have been reported regardless of the surgical method used including PTCE [3,5,33,72,78,81,104,105,117]. Older age, presence of HE immediately before surgery, shunt morphology (portoazygous), and certain breeds (especially Pugs) in dogs and lower post-operative osmolality in cats have been reported as risk factors for development of POS [51,81,104,111,115,117]. Prognosis for dogs and especially cats developing POS is poor, as the mortality rate is high, and dogs and cats that survive may have severe or permanent neurological dysfunction [3,52,107,112,117].

Another possible complication after shunt attenuation is clinically significant hypoglycemia. In a study, 7/16 dogs developed clinical hypoglycemia, and in 2/7 dogs hypoglycemia was refractory to post-operative IV dextrose supplementation [7]. Dogs with refractory hypoglycemia may respond to glucocorticoid administration (dexamethasone 0.1–0.2 mg/kg IV once). The cause of refractory hypoglycemia is unknown; however, blood glucose concentrations are not correlated with cortisol concentrations or response to adrenocorticotrophic hormone stimulation post-operatively [7].

Recurrence or persistence of clinical signs is a common complication after CPSS attenuation [32,83,87,119,120]. Possible reasons are incomplete occlusion of the CPPS with persistent shunting, suboptimal placement of the attenuation device, or development of acquired portosystemic shunts. Patients that underwent attenuation of CPSS and have persistent clinical signs and laboratory abnormalities even 5–6 months after surgery should be re-evaluated with ultrasonography or computed tomographic angiography for shunting [1]. Persistent shunting through the original ECPSS has been described in up to 21% of dogs treated with an AC and in up to 35% of dogs treated with TFB, and in up to 57% after AC placement and 3–20% after TFB placement in cats [3,45,51,88,108,120]. Despite the failure of complete attenuation, the majority of these cases were free of clinical signs. Serum BA, plasma FA, and ammonia tolerance test are the most commonly used tests in the post-operative follow-up. However, these tests are not reliable to determine shunt closure [121]. Normal ammonia concentrations do not rule out the presence of persistent shunting [121,122], as the sensitivity of fasting ammonia for the detection of residual shunting after CPSS attenuation is low (19–44%) despite the great specificity (100%) [121,122]. Increased serum BA are often found in dogs with closed ECPSS post-operatively, whereas normal serum BA concentrations are also reported in dogs with persistent shunting [121,122]. Recent studies have evaluated the usefulness of several blood tests in determining the post-operative shunt closure, such as the lidocaine/MEGX test, as well as the determination of serum hyaluronic acid and insulin-like growth factor concentrations and protein C activity [123,124,125,126]. Although they are promising, they are not easily accessible and surely, they require further evaluation to determine clinical usefulness. Post-operative advanced diagnostic imaging is still needed to confirm CPSS closure or differentiate persistent shunting through the original CPSS or due to development of acquired portosystemic shunts. However, most dogs and cats do not undergo post-operative imaging to confirm ECPSS complete attenuation unless they still have clinical signs related to liver dysfunction. Thus, failure of shunt attenuation is likely underdiagnosed. In dogs treated surgically, it is still questionable which degree of persistent shunting can be acceptable without risking recurrence of clinical signs later in life and/or decrease in life expectancy [32,54,55]. The quality of life in dogs with ECPSS improves significantly post-surgically, even in dogs with persistent shunting [127]. However, it remains unclear if further improvement can be expected after a second surgery.

**Figure 5:**
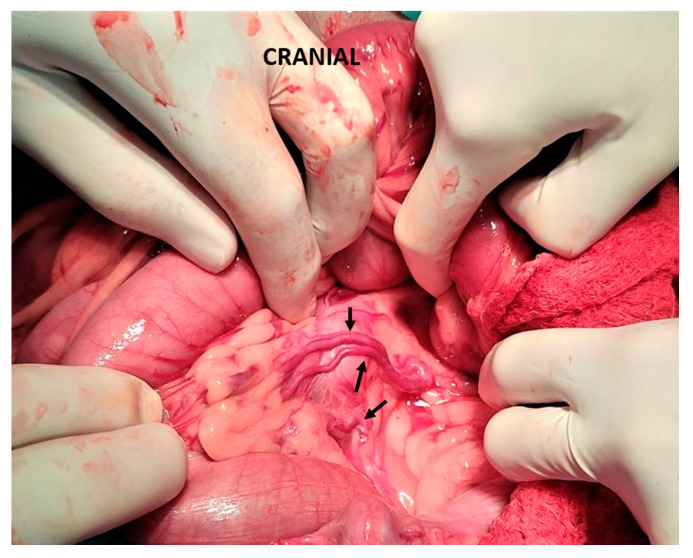
Multiple acquired shunts (arrows) that developed following a portocaval shunt attenuation using an ameroid constrictor.

Acquired portosystemic shunts develop after opening of embryonic vessels between the portal vasculature and the caudal vena cava or azygous vein. These vessels become functional when there is a pressure gradient between portal and systemic circulation. Acquired portosystemic shunts appear as multiple tortuous vessels usually around the left renal vein, the rectum, or the splenic vein but can occur anywhere in the abdomen (Figure 5) [128,129]. Acquired portosystemic shunts have been reported in 0–17.5% with AC and in 5–18% with TFB following attenuation of a ECPSS and are the result of severe PH [3,32,48,51,55,83,87,119]. Other causes of acquired portosystemic shunts are hepatic arteriovenous malformations, primary portal vein hypoplasia with PH, chronic hepatitis, and fibrosing hepatic cirrhosis [130,131]. A definitive diagnosis of acquired portosystemic shunts is made through advanced imaging or exploratory laparotomy. Acquired portosystemic shunts ligation should not be attempted as they relieve PH; treatment aims at alleviating clinical signs and is similar to medical management of CPSS [127,130,132,133,134].

## 5. Long-Term Post-operative Care

Medical management is still necessary after CPSS attenuation [1,65]. In dogs and cats that underwent attenuation of a CPSS, laboratory evaluation including liver function tests should be performed 2–3 months after surgery [1,2,52]. If the results are within normal limits and in absence of clinical signs, patients should be withdrawn from medical treatment gradually. In case of abnormal laboratory findings, patients should be rechecked 5–6 months after surgery [1]. If clinical signs and/or abnormal laboratory findings persist, patients should be evaluated with ultrasonography or computed tomographic angiography for persistent shunting [121].

## 6. Prognosis and Outcome

Many studies provide information regarding prognosis, complications, and mortality rates in dogs and cats with CPSS. However, there is a lack of consistency among them in how the outcome may be assessed. Several predictors of the outcome have been identified. Dog’s age at the time of surgery is not correlated with post-operative mortality or long-term outcome after CPSS attenuation [11,135]. In dogs with ECPSS treated surgically using AC, body weight was not associated with post-operative mortality or unsuccessful long-term outcome [3], while dogs with ICPSS weighing over 10 kg had a more favorable short-term outcome [64]. Additionally, anemia has been identified as a negative long-term prognostic factor for ECPSS or ICPSS [4,64]. Hypoproteinemia, hypoalbuminemia, and increased BUN concentration are negative short term prognostic indicators, while hypoproteinemia and low PCV are negative long term prognostic indicators for dogs with ICPSS [64]. Pre-operative leukocytosis and neutrophilia are negative long term prognostic indicators for dogs with ECPSS [3,64]. Conflicting results regarding correlation of pre-operative serum BA concentration levels with long-term outcome in dogs with ECPSS have been reported [32,54].

In dogs with CPSS undergoing suture ligation, portal pressure before ligation, during temporary occlusion, and changes in portal pressure after ligation are not correlated with long-term outcome [54,55]. As expected, dogs that tolerate complete acute ligation of the shunt have a better prognosis than those that can tolerate only partial attenuation [49,54,55,73]. In dogs with ECPSS undergoing AC placement, higher portal pressure and greater increase in portal pressure during temporary shunt occlusion are prognostic indicators for long-term negative outcome [3]. However, in a more recent study, probability of overall survival was significantly increased in dogs that had a greater portal pressure during temporary shunt occlusion [32].

Post-surgical abdominal distension in dogs undergoing ECPSS attenuation using AC has been reported as a negative short-term prognostic indicator [3]. The short-term and long-term prognosis for dogs with CPSS developing POS is poor [3,52,107,112,117]. Older dogs and cats may be more susceptible to POS; however, it is not a consistent finding among studies [51,106,117,135].

Perioperative mortality rates reported for dogs with ECPSS are 2–32% after suture ligation, 7% after AC attenuation, and 6–9% after TFB [3,11,32,43,48,49,51,55], while for dogs with ICPSS are 6–23% after suture ligation, 0–9% after AC attenuation, and 27% after TFB [11,49,51,57,64,136,137,138]. Perioperative mortality rates reported for cats with ECPSS are 4–20% after suture ligation, 0–4.5% after AC attenuation, and 0–22% after TFB [51,52,57,85,107,108,139,140].

## 7. Conclusions

Surgery is currently the preferred treatment for CPSS in order to re-establish normal portal blood flow. Several different surgical techniques, for gradual or acute attenuation of CPSS, have been proposed. Nowadays, the application of AC and TFB are the most commonly used techniques. Medical management is recommended pre-surgically for patient stabilization or if surgery is not possible. The goal of medical therapy is to decrease production and absorption of encephalopathic toxins and includes dietary adjustment and antibiotic and synthetic disaccharide administration (e.g., lactulose). Both surgical and medical management increase long-term survival; however, dogs with CPSS treated surgically live longer than those treated medically. Major complications after CPSS attenuation include POS, PH, recurrence of clinical signs, and hypoglycemia. Despite the possible complications, the prognosis after surgical attenuation of CPSS is generally good for dogs and fair for cats. Large randomized prospective studies are needed to compare the efficacy of surgical and/or medical treatments and validate outcome.

## Figures and Tables

**Table 1:** Drugs used in the management of congenital portosystemic shunts.

Antibiotics	
Metronidazole	7.5 mg/kg PO q12h
Amoxicillin	22 mg/kg PO, IV, IM, or SC q12h
Ampicillin	22 mg/kg IV q6h
Neomycin (avoid in case of intestinal bleeding, ulcerations, or renal failure; ototoxic, nephrotoxic)	20 mg/kg PO q12h
**Non-absorbable disaccharides**	
Lactulose	Orally: 2.5 to 25 mL PO q8h (two or three soft stools per day) Dogs: typically start at 0.5 mL/kg PO q8h Cats: typically start at 2.5–5 mL/cat PO q8h Rectally: cleansing enemas with water (5–10 mL/kg), followed by retention enemas (30% lactulose solution; 10–15 mL/kg), retained for 30 min to 1 h
Lactitol	0.5 to 0.75 g/kg PO q12h
**Gastroprotectants**	
**Proton pump inhibitors**	
Omeprazole	0.9–1 mg/kg PO or IV q12h
Esomeprazole	1 mg/kg PO or IV q12h
**Sucralfate**	1 g/25 kg PO q8hr

## Data Availability

Not applicable.
